# The influence of adult urine lead exposure on bone mineral densit: NHANES 2015-2018

**DOI:** 10.3389/fendo.2024.1412872

**Published:** 2024-10-02

**Authors:** Shaokang Wang, Xiaofeng Zhao, Runtian Zhou, Yuanzhang Jin, Xiaonan Wang, Xiaotian Ma, Xiangdong Lu

**Affiliations:** Department of Orthopedics, the Second Hospital of Shanxi Medical University, Taiyuan, Shanxi, China

**Keywords:** bone mineral density, urinary lead, national health and nutrition examination survey (NHANES), lead exposure, combined exposure

## Abstract

**Introduction:**

Previous studies have indicated that exposure to heavy metals related to bone health is primarily limited to some common harmful metals, and the impact of lead has not been fully understood. This study aims to explore the relationship between urine lead exposure and bone density.

**Methods:**

1,310 adults were included from the NHANES database (2015-2018), and through generalized linear regression analysis and constrained cubic spline models, the association between lead levels and total bone density as well as lumbar spine bone density was explored. The study also examined the impact of combined exposure to lead and cadmium on bone density.

**Results and conclusions:**

Urinary lead levels were significantly negatively correlated with total bone mineral density (β: −0.015; 95%CI: −0.024, −0.007) and lumbar spine bone mineral density (β: −0.019; 95%CI: −0.031, −0.006). Compared to the lowest three quartiles of lead levels, the adjusted odds ratios for T3 changes in total bone mineral density and lumbar spine bone mineral density were 0.974 (95%CI: 0.959, 0.990) and 0.967 (95%CI: 0.943, 0.991), indicating a significant negative trend. Further analysis with constrained cubic spline models revealed a non-linear decreasing relationship between urinary lead and total bone mineral density as well as lumbar spine bone mineral density. Stratified analyses suggested that the relationship between urinary lead levels and bone mineral density might be significantly influenced by age, while gender showed no significant impact on the relationship. Moreover, combined exposure to lead and cadmium was found to be associated with decreased bone mineral density, emphasizing the potential synergistic effects between lead and cadmium on bone health. However, the specific mechanisms of lead and its effects on different populations require further comprehensive research. This study provides valuable insights for further exploration and development of relevant public health policies.

## 1 Background

Osteoporosis is a metabolic bone disease characterized by changes in bone microstructure and decreased bone mineral density (BMD) (1). Bone density is an important indicator for evaluating bone health, with the two most common sites for measuring bone density being the whole body and the lumbar spine (2). Factors leading to decreased bone density include genetics (3), metabolism (4), and nutrition (5). According to recent evidence, decreased bone density may be related to environmental toxin exposure (6, 7), and heavy metals such as lead (Pb) may be associated with osteoporosis and related fractures (8, 9).

As a typical heavy metal, lead has received much attention due to its harmful effects on human health. The role of lead exposure in human biology is intricate and far-reaching, impacting not only the endocrine system by disrupting thyroid and sex hormone levels but also exerting significant effects on the nervous, immune systems, and reproductive health. As an endocrine-disrupting compound (EDC), lead binds to estrogen and androgen receptors, mimicking estrogenic effects and obstructing androgen actions, thus disturbing hormonal balance (10). This action is not only linked to thyroid dysfunction but also to reproductive health issues, particularly in males, where the association between lead exposure and infertility is increasingly evident. Lead exposure further damages reproductive capabilities through the induction of reactive oxygen species (ROS), cell apoptosis, local necrosis, immunosuppression, and mutagenic stimulation, negatively impacting the male reproductive system and potentially leading to azoospermia (11).

Moreover, lead exposure significantly impairs cognitive and behavioral development in children, correlating with decreased IQ, alterations in neurotransmitter levels, and reduced cognitive and behavioral scores (12–15). In terms of immune function, lead exposure is associated with altered levels of pro-inflammatory cytokines in children, potentially triggering a cascade of health issues spanning neurological, respiratory, cardiovascular, reproductive, and renal systems (16). More alarmingly, lead and its compounds are classified by the International Agency for Research on Cancer (IARC) as probable human carcinogens, indicating a potential cancer risk associated with long-term lead exposure (17). Although the organ toxicity of lead has been widely studied, research on the impact of lead on human bone health is limited.

More than 90% of lead in the human body is found in the bones (18). Lead has strong cytotoxicity, affecting osteoblasts, osteoclasts, and chondrocytes (19). An vitro studies have shown that lead can replace calcium in hydroxyapatite crystals and has a higher affinity for bone sialoprotein than calcium (20). Many animal studies have reported that lead exposure is associated with pathological processes in bone, resulting in decreased bone density and strength (21). In the United States, studies on elderly individuals have shown a significant negative correlation between blood lead levels and osteoporosis, particularly among Caucasian subjects (22). Furthermore, a study conducted in Taiwan found that adults, especially females, with higher urinary lead levels may have an increased risk of osteopenia and osteoporosis (23). Other studies have indicated that lead exposure is associated with femoral and spinal bone density in premenopausal women in the United States (24, 25), and lead and manganese exposure have been found to have a synergistic effect on bone density (8). The toxic effects of lead on bone density in different bone sites vary among children and adolescents, and there are differences in various age groups, genders, and levels of exposure (26). Overall, the research on the effects of normal lead exposure on bone density in adults is still limited, and further systematic studies are needed to obtain accurate conclusions.Therefore, we utilized data from the 2015-2018 National Health and Nutrition Examination Survey (NHANES) database to investigate the correlation between urinary lead levels and bone density in a representative sample of adults aged 20 and above in the United States.

## 2 Subjects and methods

### 2.1 Design

The research data is derived from the NHANES database and is a cross-sectional study. All analyses were performed under logarithmic transformation and statistical analysis was conducted using multiplicative interaction models and generalized linear regression models. For other continuous variables, differences between groups were calculated using generalized linear regression models. Weighted chi-square tests were used for categorical variables.

### 2.2 Time and Location

The study selected information from the US NHANES database from January 2015 to December 2018, and the samples were taken from the general population of the United States.

### 2.3 Subjects

**Figure 1:**
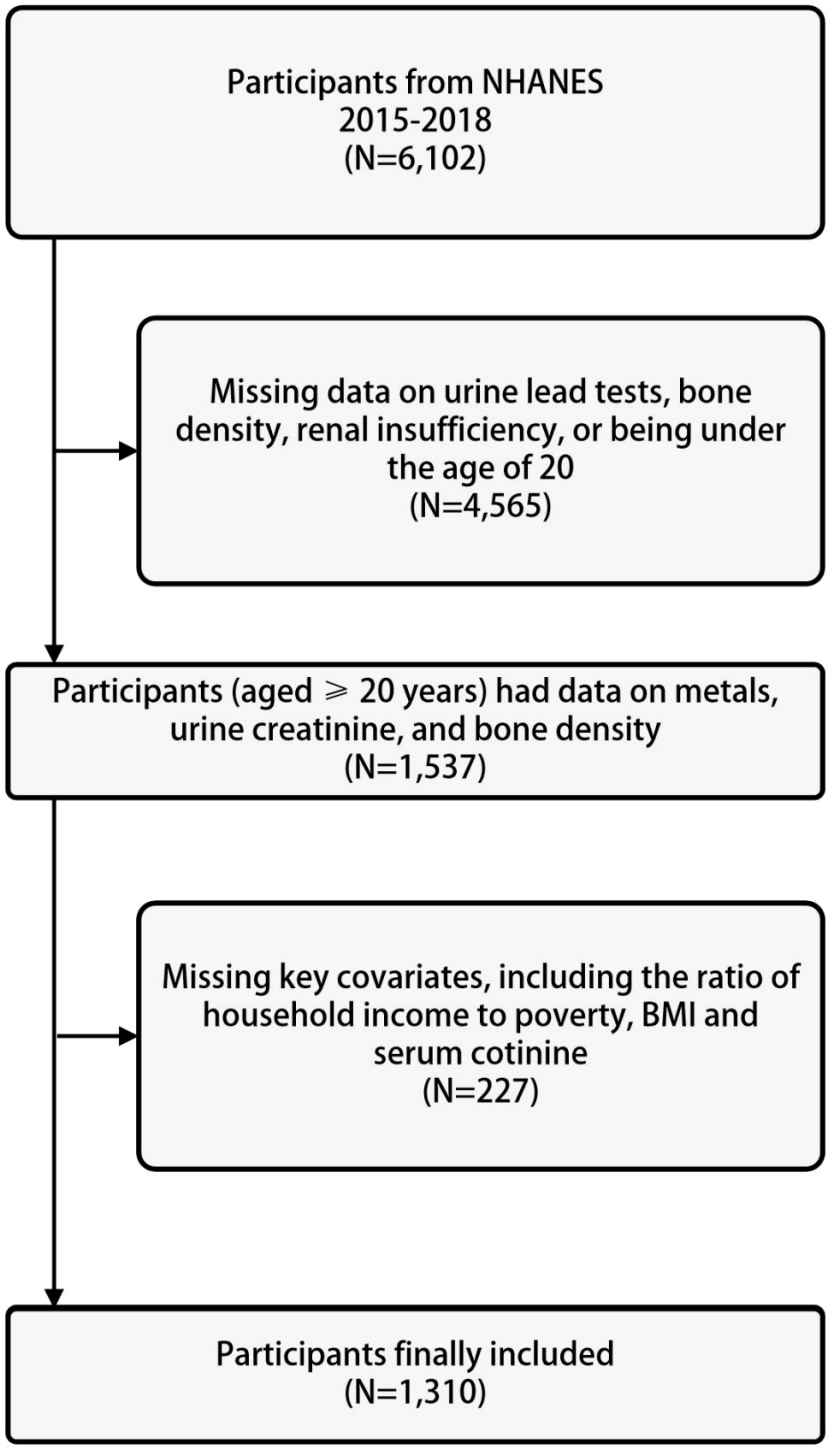
Baseline characteristics of participants.

The data for the study was derived from the US NHANES database. The NHANES database collects nutritional and health information from the general population of the United States and is a cross-sectional study. The NHANES database uses a large-scale, multi-stage complex sampling method, with non-repetitive sampling population, abundant sample size, and good representativeness. The study was approved by the Ethics Review Committee of the National Center for Health Statistics (NCHS), with ethics protocol numbers Continuation of Protocol #2011-17 (2013-2016) and Protocol #2018-01 (2017-2020), and written informed consent was obtained from each participant. The study subjects were selected from data spanning four years, from 2015 to 2018. Among the participants who underwent urinary metal testing from 2015 to 2018, a total of 6102 individuals were included. After excluding individuals with missing data on urine lead, bone density, renal insufficiency, or age less than 20 years (n=4565), 1537 participants were selected. Further exclusions were made for individuals with missing information on basic covariates including poverty-income ratio, body mass index, serum cotinine, and serum 25(OH)D (n=227), resulting in a final analytical sample of 1310 participants, as shown in **Figure 1**.

### 2.4 Methods

The selection of covariates is based on previous literature (27). The final covariates include age, gender (male and female), race/ethnicity (Mexican American, Other Hispanic, Non-Hispanic White, Non-Hispanic Black, Other), body mass index (kg/m2), poverty income ratio (<1, ≥1), education level (less than high school, high school or equivalent, higher than high school), serum cotinine (≥10ng/mL, 1-9.9ng/mL, and <1ng/mL), physical activity (<10 minutes/week and ≥10 minutes/week), serum 25(OH)D, thyroid disease (yes or no), diabetes (yes or no), and hypertension (yes or no).

Urine metal determination: Urine samples were collected and stored at -70°C, then transported to the National Center for Environmental Health for testing. Urine lead concentration was measured using inductively coupled plasma mass spectrometry (ICPMS). The laboratory procedures are described in detail on the NHANES website. The metals included in this study were detectable in over 99% of the participants. The metal levels in urine were calibrated with urine creatinine and expressed as μg/g creatinine.At a dedicated mobile examination center, total bone density and lumbar spine bone density were measured using the Hologic QDR 4500A fan-beam dual-energy X-ray absorptiometry (Hologic, Inc., Bedford, Massachusetts). For more detailed information on bone density assessment, please visit the NHANES website.

### 2.5 Statistical analysis

Due to skewed distributions, both bone density and urine lead concentration underwent natural logarithm (ln) transformation. Descriptive analysis was conducted on the participants’ basic demographic characteristics and bone density. Continuous variables were expressed as means and standard deviations (± s) or percentiles, while categorical variables were expressed as frequencies (proportions).

Generalized linear regression was used to evaluate the correlation between individual urine lead and bone density, treating each metal as a continuous exposure variable. The transformed regression coefficient represents the percentage change in bone density with a doubling of urine metal levels, using the following formula: (e(ln2 × β) - 1) × 100%. To further explore the relationship between urine lead and bone density, the generalized linear regression model treated urine lead concentration as tertiles. The percentage change in bone density associated with urine metal tertiles was estimated as (eOR - 1) × 100%. Restricted cubic splines (RCS) were used to assess the dose-response relationship between urine lead and bone density. The RCS model included three knots: the 25th, 50th, and 75th percentiles of the transformed metal concentration.

Stratified analysis by gender and age was conducted, followed by multiplicative interaction analysis. Generalized linear regression was used to further evaluate the combined effect of urine lead and urine cadmium exposure. Participants were divided into low exposure and high exposure groups based on the median levels of the metals. The group with low exposure to both metals was considered as the reference group. The percentage change in bone density for the exposed group was estimated as (eOR - 1) × 100%.

All analyses were performed using R (version 4.2.3), with statistical significance set at P<0.05 (two-tailed). Generalized linear regression analysis and RCS models were implemented using the “ggplot2” and “rms” packages, respectively.

## 3 Results

### 3.1 Baseline characteristics

The study included a total of 1310 subjects, with mean ages and body mass indexes of 39.5 ± 11.2 years and 29.0 ± 6.9 kg/m^2^ , respectively. Among the study population, 642 individuals (49.0%) were male, 777 individuals (59.3%) had received higher education (beyond high school), 388 individuals (29.6%) were non-Hispanic white, 1069 individuals (81.6%) were at or above the poverty line, 915 individuals (69.8%) were non-smokers, 86 individuals (6.6%) were informed of thyroid issues, 101 individuals (7.7%) had diabetes, and 275 individuals (21.0%) had hypertension. The median serum 25(OH)D, total bone density, and lumbar spine bone density were 56.7 (42.0, 73.7) nmol/L, 1.11 (1.04, 1.18) g/cm^2^, and 1.02 (0.93, 1.12) g/cm^2^, respectively. See **Table 1** for details.

**Table 1:** Characteristics of included participants from NHANES 2015–2018 (N = 1310).

Characteristics	Means ± SDs/N (%)/median (25th, 75th)
Age	39.5± 11.2
Sex	
Male	642 (49.0)
Female	668 (51.0)
Race/ethnicity	
Mexican American	226(17.3)
Other Hispanic	156(11.9)
Non-Hispanic White	388(29.6)
Non-Hispanic Black	259(19.8)
Other Race	281(21.5)
BMI(kg/m^2^)	29.0 ± 6.9
Family PIR	
<1	241(18.4)
≥1	1069(81.6)
Education	
Under high school	238(18.2)
High school or equivalent	295 (22.5)
Above high school	777(59.3)
Serum cotinine (ng/mL)	
<1.0 ng/mL	915 (69.8)
1.0-9.9 ng/mL	43(3.3)
≥10 ng/mL	352(26.9)
Physical activity (n/%)	
<10 minutes/week	987 (75.3)
≥10 minutes/week	323(24.7)
Thyroid disease (n/%)	
Yes	86 (6.6)
No	1224 (93.4)
Hypertension (n/%)	
Yes	275 (21.0)
No	1035(79.0)
Diabetes (n/%)	
Yes	101 (7.7)
No	1209(92.3)
Serum 25(OH)D (M(Q_25,_Q_75_),nmol/L)	56.7(42.0,73.7)
Total bone density (M(Q_25,_Q_75_),g/cm²)	1.11(1.04,1.18)
Lumbar bone density (M(Q_25,_Q_75_),g/cm²)	1.02 (0.93, 1.12)

### 3.2 Distribution of urinary lead levels

The geometric mean of urine lead concentration corrected for urinary creatinine was 3.918 μg/g creatinine. The median urine lead concentration was 2.826 μg/g creatinine, with an interquartile range (IQR) of 1.785-4.580 μg/g creatinine, and the standard deviation of urine lead was 4.0927 μg/g creatinine.

### 3.3 Correlation between urinary lead exposure and bone density

**Figure 2:**
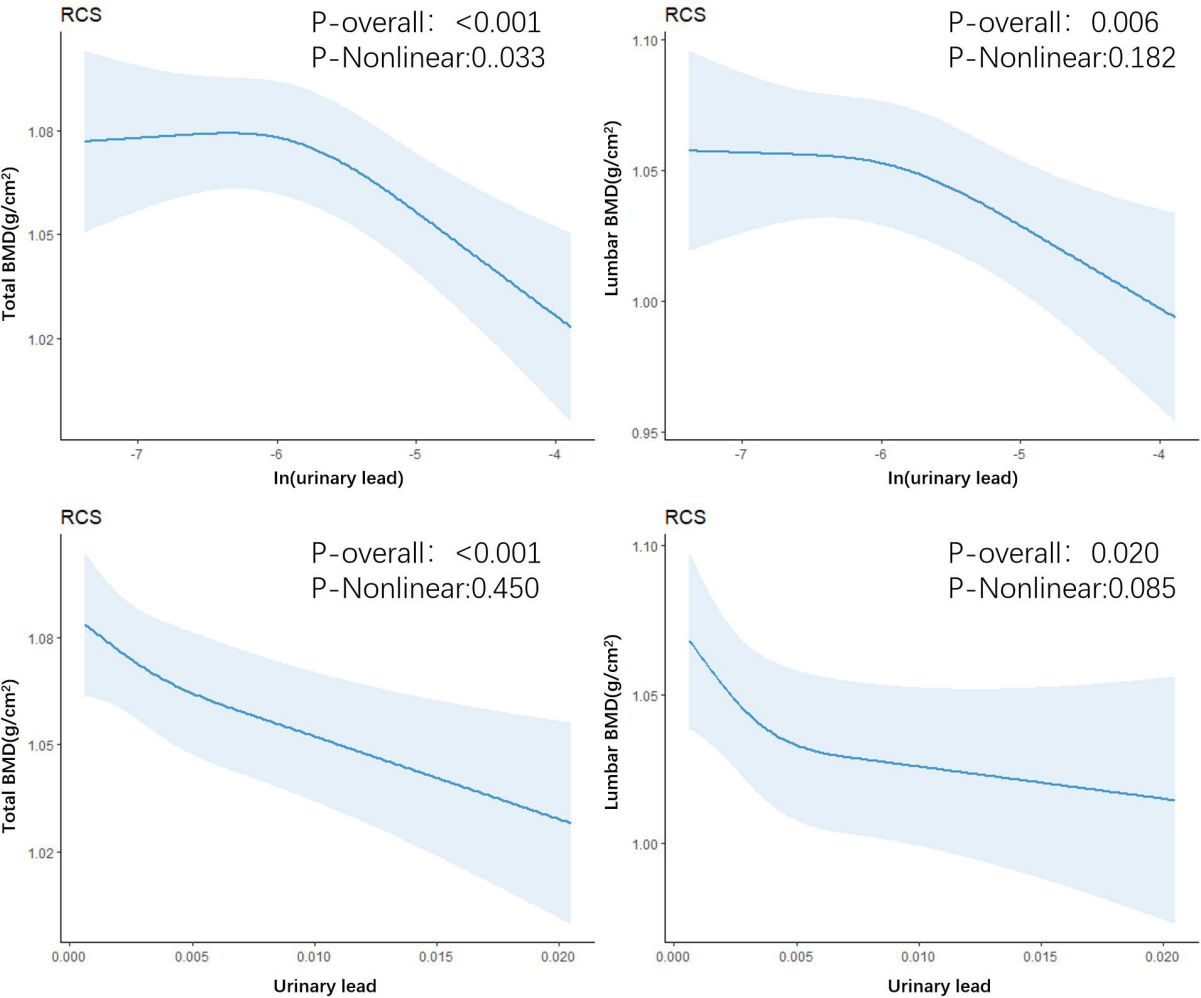
Restricted cubic spline regression of urinary lead with total bone density and lumbar spine bone density.

In the fully adjusted model, a significant negative correlation was observed between urinary lead levels and total bone density (β: -0.015; 95%CI: -0.024, -0.007) as well as lumbar spine bone density (β: -0.019; 95%CI: -0.031, -0.006). Furthermore, in the multivariable adjusted model, compared with the lowest tertile of lead level, the odds ratios (ORs) (95%CI) of total bone density at T2 and T3 levels were 0.997 (0.982, 1.011) and 0.974 (0.959, 0.990), respectively, when introducing the tertiles of urinary lead concentration. Similarly, compared with the lowest tertile of lead level, the ORs (95%CI) of lumbar spine bone density at T2 and T3 levels were 0.999 (0.978, 1.021) and 0.967 (0.943, 0.991) (**Table 2**), respectively. This indicates a significant negative correlation between total bone density and lumbar spine bone density at the highest tertile of urinary lead concentration. It is worth noting that the relationship between these bone density indicators and moderate levels of urinary lead concentration was not significant. This result emphasizes the different effects of urinary lead concentrations on bone density at different levels. A restricted cubic spline showed a nonlinear relationship between urinary lead and total bone density as well as lumbar spine bone density (**Figure 2**).

**Table 2:** Associations between urinary lead exposure and BMDs.

Urinary lead (ug/mg creatinine)	Percentage change (95% CI) in BMDs			
	Total BMD	P	Lumbar BMD	P
Per 100% increase	−0.015 (−0.024, −0.007)	<0.001	−0.019 (−0.031, −0.006)	0.004
Tertiles				
T1(< 0.216)	Reference		Reference	
T2(0.216–0.382)	0.997 (0.982, 1.011)	0.668	0.999 (0.978, 1.021)	0.946
T3(≥ 0.382)	0.974 (0.959, 0.990)	0.002	0.967 (0.943, 0.991)	0.007

These models were adjusted for factors such as age, sex, body mass index, race, family income-to-poverty ratio, education, serum cotinine levels, physical activity, serum 25(OH)D, thyroid disease, hypertension, and diabetes.

In a multivariable adjusted model, gender-stratified analysis revealed that the β values (95% CI) of urinary lead levels and total bone density in men and women were −0.012 (−0.025, 0.0004) and −0.021 (−0.033, −0.010), with corresponding P values of 0.059 and <0.001, respectively. For women, there was a significant negative correlation between urinary lead levels and total bone density, with a β value of −0.021, a 95% CI not including zero, and P value <0.001. However, in men, although the β value was negative, the P value did not reach significance. As for the relationship between urinary lead levels and lumbar bone density, the β values (95% CI) in men and women were −0.021 (−0.033, −0.010) and −0.022 (−0.040, −0.004), with P values of 0.089 and 0.015, respectively. Urinary lead levels were significantly negatively correlated with lumbar bone density in both men and women. However, in men, the P value was 0.089, which did not reach significance, while in women, the P value was 0.015, indicating a higher level of significance (**Table 3**).

**Table 3:** Interaction of sex and age on the relationship between urinary lead levels and total BMD and lumbar BMD.

Urinary lead (ug/mg creatinine)		Percentage change (95% CI) in BMDs				
	Total BMD	P	P_int_	Lumbar BMD	P	P_int_
**Sex**			0.241			0.380
Male	−0.012(−0.025, 0.0004)	0.059		−0.016 (−0.034, 0.002)	0.089	
Female	−0.021 (−0.033, −0.010)	<0.001		−0.022 (−0.040, −0.004)	0.015	
**Age**			0.034			0.045
<39(Median)	-0.009 (-0.021, 0.003)	0.138		−0.006 (−0.022, 0.011)	0.509	
≥39(Median)	-0.023(-0.035, -0.010)	<0.001		−0.031 (−0.051, −0.012)	0.001	

These models were adjusted for factors such as age, sex, body mass index, race, family income-to-poverty ratio, education, serum cotinine levels, physical activity, serum 25(OH)D, thyroid disease, hypertension, and diabetes.

Overall, the relationship between urinary lead levels and total bone density as well as lumbar bone density was significant in women and tended towards a negative correlation in men but was not significant. However, the overall bone P_int_ for gender stratification was 0.241, and the lumbar bone density P_int_ was 0.380, indicating no significant interactive effects, showing that gender had no significant impact on the relationship between urinary lead levels and bone density.

Similarly, in a multivariable adjusted model, stratified analysis based on the median age (39 years) revealed that the β values (95% CI) of urinary lead levels and total bone density in the low-age and high-age groups were −0.009 (−0.021, 0.003) and −0.023 (−0.035, −0.010), with corresponding P values of 0.138 and <0.001, respectively. Regarding total bone density, the relationship between urinary lead levels and bone density was not significant in the low-age group (P value = 0.138), while in the high-age group, there was a significant negative correlation between urinary lead levels and total bone density (P value <0.001). As for the relationship between urinary lead levels and lumbar bone density, the β values (95% CI) in the low-age and high-age groups were −0.006 (−0.022, 0.011) and −0.031 (−0.051, −0.012), with P values of 0.509 and 0.001, respectively. In terms of lumbar bone density, the relationship between urinary lead levels and bone density was similarly not significant in the low-age group (P value = 0.509), whereas there was a significant negative correlation between urinary lead levels and lumbar bone density in the high-age group (P value = 0.001).

Furthermore, the overall bone P_int_ for age stratification was 0.034, and the lumbar bone density P_int_ was 0.045. These data suggest that the relationship between urinary lead levels and bone density may be significantly influenced by age, and in the high-age group, there is a stronger correlation between increasing urinary lead levels and decreasing bone density.

### 3.4 Joint effect analysis

Further evaluates the combined effects of lead and cadmium exposure on total bone density and lumbar spine bone density. The groups with low exposure levels to both metals were considered as the reference group. The odds ratios (OR) for total bone density in the low-cadmium high-lead exposure group, high-cadmium low-lead exposure group, and high-cadmium high-lead exposure group were 0.991 (0.976, 1.008), 1.002 (0.985, 1.020), and 0.984 (0.968, 1.001) respectively, with p-values of 0.337, 0.806, and 0.071. The odds ratios (OR) for lumbar spine bone density in the low-cadmium high-lead exposure group, high-cadmium low-lead exposure group, and high-cadmium high-lead exposure group were 0.989 (0.966, 1.014), 1.000 (0.975, 1.026), and 0.968 (0.945, 0.993) respectively, with p-values of 0.387, 0.999, and 0.012 (**Table 4**). Through the high-cadmium high-lead exposure group, it is demonstrated that the combined exposure to cadmium and lead has a negative impact on bone density, and this effect is statistically significant.

**Table 4:** The impact of lead and cadmium combined exposure on BMDs.

Metals	N	Total BMD Percent change (95% CI)	P	Lumbar BMD Percent change (95% CI)	P
cadmium - lead					
Low Cd + low Pb	427	Reference		Reference	
Low Cd + high Pd	228	0.991(0.976,1.008)	0.337	0.989(0.966,1.014)	0.387
High Cd + low Pb	228	1.002(0.985,1.020)	0.806	1.000(0.975,1.026)	0.999
High Cd + high Pd	427	0.984(0.968,1.001)	0.071	0.968(0.945,0.993)	0.012

These models have been adjusted for factors such as age, gender, body mass index, race, family income to poverty ratio, education, serum cotinine levels, physical activity, serum 25(OH)D, thyroid disorders, hypertension, and diabetes.

## 4 Discussion

In this study, we investigated the relationship between urinary lead exposure and bone density during the NHANES survey period from 2015 to 2018. Overall, our study results showed that urinary lead exposure was associated with reduced total bone density and lumbar spine bone density. In stratified analysis, it was also found that urinary lead levels were correlated with decreased bone density, and the relationship between urinary lead levels and bone density may be significantly influenced by age with no significant gender effect observed. The combined effect of lead and cadmium was found to be related to decreased bone density.

The results of this study showed a negative correlation between urinary lead exposure levels and bone density. To our knowledge, this is the largest epidemiological research report on lead exposure and bone density across a wide age range. Lead is highly toxic and lead poisoning can cause damage to the nervous system and brain function. There have been few studies on the impact of lead on bone health. A report from Sweden found no association between adult lead exposure and bone density (28). The potential mechanism by which elevated lead levels cause bone disease is not clear. *In vitro* studies have shown that lead can exchange calcium in hydroxyapatite crystals, with higher affinity for osteocalcin than calcium (20), and inhibit the activation of vitamin D and dietary calcium absorption (29). However, more investigations are needed to validate our research results and elucidate the specific mechanisms behind the reduction of bone density with lead exposure.

Plasma lead concentration is difficult to accurately measure because of its low concentration and susceptibility to contamination (30). Whole blood lead levels are usually used as a biological marker of lead exposure because over 99% of lead is bound to red blood cells. However, due to the saturation effect of lead-binding sites in red blood cells, male blood lead levels are higher than female levels, while urinary lead levels show no significant difference between genders (24). Urinary lead is considered an alternative indicator reflecting plasma lead levels, as lead is mainly filtered through the glomerulus and excreted in urine. To accurately reflect urinary lead excretion, adjustments for urine dilution need to be considered. This study used urinary lead as a biological marker of lead exposure.

One finding in our study is the non-linear negative correlation between urinary lead levels in adults and total bone density and lumbar bone density. We found a stronger association between increasing urinary lead levels and decreasing bone density in the older age group (≥39 years old). This differs from previous studies in adults (8), but the reasons are still unclear. It could be due to variations in hormone levels at different ages. Further research is needed to investigate the differences in bone remodeling, absorption, and formation capabilities in different bone sites under various levels of lead exposure and in different age groups. We found that urinary lead concentration has different effects on bone density at different levels, and as urinary lead concentration increases, the decrease in bone density shows an initial rapid decline followed by a slower decline. The differences in lead’s impact on bone density may be attributed to the complex mechanisms of lead metabolism in bone at different levels. Previous studies have found that long-term lead exposure at low levels (blood levels <10μg/dL) inhibits the Wnt signaling pathway and leads to decreased bone density in adult rats, while increased bone mass has been observed in mice exposed to high levels of lead, which inhibits the ability of osteoclasts to reabsorb bone mass (31). This may suggest the need for greater attention to the effects of lead exposure on bone health in older age groups.

In our study, there was a significant relationship between urinary lead levels and total bone density and lumbar bone density in females, while in males, the correlation tended to be negative but not significant. In gender-stratified analyses, the P interaction values were 0.241 for total bone density and 0.380 for lumbar bone density, indicating that gender does not significantly affect the relationship between urinary lead levels and bone density. The negative association between lead exposure levels and bone density in adult women is consistent with previous studies, which found an association between lead exposure and decreased bone density in premenopausal women (32). However, another study of 50-70-year-old women also found a non-significant correlation (33). Even at low levels, lead can affect follicles in mice (34), which are the primary source of endogenous estrogen. Due to the decrease in estrogen, women experience rapid bone loss in the first 5-10 years after menopause (35). Therefore, we speculate that lead exposure induces a decrease in bone density by suddenly lowering estrogen levels. No association between lead concentration and bone density was observed in postmenopausal women because their ovaries no longer produce endogenous estrogen. The lack of significant correlation in males may be due to the fact that males have higher peak bone mass than females in early adulthood.

In this study, the combined exposure of cadmium and lead has a negative impact on bone density, and this impact is statistically significant, emphasizing a potential synergistic effect of these two metals on bone density. The production of reactive oxygen species induced oxidative stress is an important mechanism of lead and cadmium toxicity, which may be crucial for bone metabolism (36). In the real world, humans are simultaneously exposed to multiple heavy metals, which interact with each other. Further research is needed to validate this finding and explore its potential mechanisms in the future.

The relationship between urinary lead exposure and bone density is intricate, encompassing interactions with bone metabolism, endocrine regulation, oxidative stress, and interplays with age, gender, and metabolic status. When evaluating bone density risk, a holistic consideration of multiple risk factors, including urinary lead levels, elements of metabolic syndrome (e.g., abdominal obesity, hypertension, glucose abnormalities), and demographic factors, is essential. The potential interplay between urinary lead exposure and components of metabolic syndrome deserves attention (37). For example, certain aspects of metabolic syndrome may influence the body’s lead handling, affecting lead’s absorption, distribution, and excretion, and thus altering the lead burden in the body, which can have implications for bone density. Concurrently, lead exposure could exacerbate metabolic syndrome risks through its impacts on metabolic functions, such as insulin resistance and lipid metabolism, indirectly affecting bone health (38).

This study has several key strengths. First, it reports an epidemiological study of the maximum range of age groups with a negative correlation between lead exposure and bone density. Additionally, objectively measured urinary lead levels were used as a biomarker reflecting lead status. Furthermore, this study also conducted stratified analyses, yielding more stable results. However, the study also has some limitations. First, our study is a cross-sectional study, and further longitudinal research is needed to investigate more accurate causal relationships. Second, potential residual confounding factors such as genetics, diet, and other environmental chemicals were not fully considered. Lastly, during the continuous physiological process of bone remodeling, nearly 10% of bone is rebuilt each year (39), which this study cannot reflect in long-term bone health.

Our study has unveiled a nonlinear negative correlation between urinary lead exposure and bone density, along with variations in this association across different age and gender groups. This finding holds significant implications for clinical practice and public health interventions. At the clinical level, healthcare professionals should recognize lead exposure as a potential risk factor for osteoporosis, particularly in older women. Public health strategies should encompass educating the public on measures to reduce lead exposure, such as abstaining from lead-containing products, ensuring the safety of drinking water, and enforcing stricter regulations on industrial emissions, all aimed at safeguarding public bone health.

## Data Availability

The original contributions presented in the study are included in the article/supplementary material. Further inquiries can be directed to the corresponding author.
